# Retrieval of Size Distribution and Concentration of Au-Ag Alloy Nanospheroids by Spectral Extinction Method

**DOI:** 10.3390/ma15051778

**Published:** 2022-02-26

**Authors:** Yuxia Zheng, Paerhatijiang Tuersun, Remilai Abulaiti, Dengpan Ma, Long Cheng

**Affiliations:** Key Laboratory of Mineral Optical Functional Materials and Application, School of Physics and Electronic Engineering, Xinjiang Normal University, Urumqi 830054, China; dearprof@126.com (Y.Z.); ramila0920@163.com (R.A.); mdp5201314@mail.ustc.edu.cn (D.M.); chenglong3604@mail.ustc.edu.cn (L.C.)

**Keywords:** spectral extinction method, composite nanoparticles, localized surface plasmon resonance, T-matrix, Tikhonov regularization, inverse problem

## Abstract

In order to monitor the synthesis processes or characterize nanoparticles for application, a new method that allows in situ determination of the two-dimensional size distribution and concentration of Au-Ag alloy nanospheroids, based on their extinction spectrum, is developed. Non-negative Tikhonov regularization and T-matrix method were used to solve the inverse problem. The effects of the two-dimensional size steps, wavelength range, and measurement errors of extinction spectrum on the retrieval results were analyzed to verify the feasibility and accuracy of the retrieval algorithm. Through comparative analysis, the size steps and wavelength range that make the retrieval error smaller are found. After adding 0.1% random noise to the extinction spectrum, a small variation in the retrieval error of the mean size is observed. The results showed that the error of the mean size is smaller than 2% and the error of the concentration is smaller than 3%. This method is simple, fast, cheap, nondestructive, and can be done in situ during the growth process of nanoparticles.

## 1. Introduction

At the nanoscale, when incident light set at specific wavelength interacts with metal nanoparticles, a local surface plasmon resonance phenomenon (LSPR) will occur. LSPR phenomenon enhances the radiative properties of the nanoparticles and consequently the nanoparticles absorb and scatter the incident light strongly. The resonance wavelength can be tuned from near-ultraviolet (NUV) to near-infrared (NIR) [1,2]. Based on these unique optical properties, metal nanoparticles have a wide range of applications in many fields, such as bioimaging [3], photothermal therapy [4], and biosensing [5,6].

With the rapid development of nanomaterials preparation technology, the research of monometallic nanomaterials can no longer meet the needs of some applications. Among the precious metal nanomaterials, Au-Ag bimetallic alloy nanomaterials have become one of the hot research topics [7,8,9]. Au nanoparticles have excellent chemical stability and biocompatibility and Ag nanoparticles have high refractive index sensitivity and surface-enhanced Raman scattering activity. Thus, the development of Au-Ag alloy nanoparticles has attracted widespread attention based on the inherent advantages of its two component elements. For instance, researchers have found that the solar photothermal conversion performance of Au-Ag alloy nanoparticles is higher than that of the pure Au nanoparticles [10]. Au-Ag alloy nanourchins exhibited higher specific surface area and better conductivity [11]. Hollow Au-Ag alloy nanorices showed good anti-oxidation and have broad application prospects in surface-plasmon-related fields [12]. The LSPR properties of metal nanoparticles are related to size, shape, concentration, and other factors of the particle. Among them, the size and concentration are two important parameters. They determine the particle’s optical properties and behaviors in practical applications [13,14]. Non-spherical nanoparticles have better optical properties compared to spherical nanoparticles. Therefore, it is crucial to establish effective methods to accurately measure the particle size and concentration of non-spherical Au-Ag alloy nanoparticles.

At present, the more accurate method for measuring the size of nanoparticles is the electron microscopy including scanning electron microscope and transmission electron microscope. However, electron microscope measurement is costly, time-consuming, and cannot achieve real-time monitoring, nor it is possible to determine the concentration of nanoparticles [15]. Among many size measurement methods, the spectral extinction method has attracted significant attention by its relative simplicity and its ability to obtain the size and the concentration of the nanoparticles simultaneously [16]. Khlebtsov et al. [17,18] retrieved the aspect ratio distribution of nanorods by fitting their extinction spectrum and depolarizing scattering spectrum. Additionally, they used the width of nanorods measured by transmission electron microscope as a priori information. With the estimation of mean diameter and end-cap of Au nanorods as a priori information, Xu et al. [19] measured the aspect ratio distribution of Au nanorods by spectral extinction method. In the above-mentioned studies, only the aspect ratio distribution was retrieved but the other two-dimensional size distribution measured by microscopic imaging could not be obtained at the same time, nor could it be measured in real-time.

In this paper, an improved inverse algorithm is used to quickly retrieve the two-dimensional size distribution (aspect ratio distribution and length distribution) and concentration of the polydisperse Au-Ag alloy nanospheroids in aqueous solution. The current study provides a new method for measuring the two-dimensional size distribution and concentration of Au-Ag alloy nanospheroids.

## 2. Theoretical Methods

**Figure 1:**
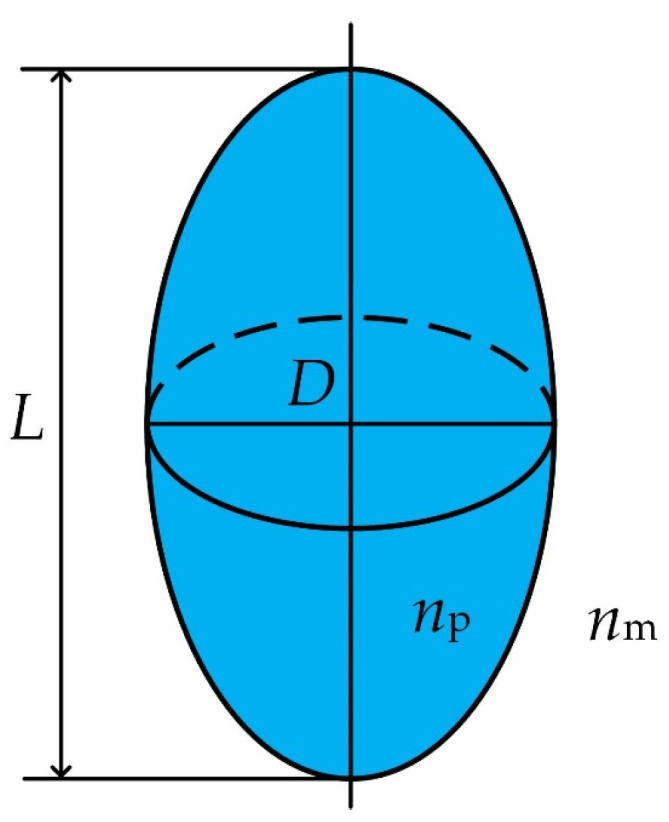
Geometry of the Au-Ag alloy nanospheroid. Here, *L* and *D* represent the length and the diameter of the alloy nanospheroid, respectively.

For the Au-Ag alloy nanospheroid, the length (*L*) and the diameter (*D*) are usually used to describe its geometric shape, as shown in Figure 1. The ratio between the length *L* and the diameter *D* is called the aspect ratio (*AR*), i.e., *AR* = *L*/*D*. When *AR* < 1 or *AR* > 1, it is called oblate ellipsoid, or prolate ellipsoid, respectively. In this work, we choose the prolate ellipsoid as a research object.

When a beam of light passing through a medium encounters a nanoparticle, scattering and absorption occurs causing reduction of the beam intensity. In the spectral extinction method, the degree of attenuation is usually expressed in terms of absorbance *A* [19]. The absorbance *A* of the nanoparticle systems in the NUV to NIR band can be accurately measured using a spectrophotometer. Under the condition of single scattering, i.e., when the interparticle distance is three times the particle diameter, the absorbance *A* of the monodisperse Au-Ag alloy nanospheroids particle systems can be expressed as [19]

(1) $$A{=\log}_{10}\left(I_{i}/I_{t}\right)=\frac{lN}{\ln\left(10\right)}C_{\mathrm{ext}}\left(\lambda ,AR,L,n_{p},n_{m}\right),$$

where *I*_i_ is the incident light intensity, *I*_t_ is the transmitted light intensity, *l* is the optical path defined as the distance of the light beam passing through the nanoparticle systems, *N* is the nanoparticle concentration, and *C*_ext_ is the extinction cross section area of a single Au-Ag alloy nanospheroid particle. *C*_ext_ is calculated using the T-matrix method [20,21]. In general, *C*_ext_ is a function of the incident light wavelength *λ*, the particle aspect ratio *AR*, the length *L*, the particle refractive index *n*_p_, and the medium refractive index *n*_m_. When calculating the refractive index of the nanoparticles, the alloy dielectric function model established by Rioux et al. [22] is used. The dimension correction of the dielectric function model is proceeded according to the shortening effect of mean free paths of free electron in metal nanoparticles. In this study, we choose the Au-Ag alloy with equal mole fraction ratio, which has the complete mixture of components. Here, we have to mention that the data used in this work about the refractive indices of the particles with ambient environment of aqueous solution at 20 °C are from reference [23].

In practical applications, since there are often no ideal monodisperse particle systems, the polydisperse particle systems are approximately considered equivalent to the monodisperse particle systems. When the polydisperse nanoparticle systems satisfy the single scattering condition, its absorbance *A* can be expressed as [19]

(2) $$A\left(\lambda \right)=\log_{10}\left(I_{i}/I_{t}\right)=\frac{lN}{\ln\left(10\right)}\sum_{j=1}^{J}\sum_{i=1}^{I}p\left(AR_{j},L_{i}\right)\;C_{\mathrm{ext}}\left(\lambda ,AR_{j},L_{i},n_{p}(\lambda ,AR_{j},L_{i}),n_{m}(\lambda )\right),$$

where the absorbance *A* contains the information of the aspect ratio *AR*, the length *L,* and the particle concentrations *N*. Here, *p*(*AR_j_*, *L_i_*) is the percentage (probability) of the number of nanospheroids related to the ratio *AR_j_* and to the length *L*_i_ in the whole particle systems. *p*(*AR_j_*, *L_i_*) must satisfy the normalization condition:

(3) $$\sum_{j=1}^{J}\sum_{i=1}^{I}p\left(AR_{j},L_{i}\right)\;=\;1,$$

In Equation (2), the retrieval problem of the two-dimensional size distribution and the concentration of the polydisperse particle systems can be regarded as a problem of solving a linear equation system. The linear equation relationship can be written as

(4) CP=A,

where the expressions for *C*, *P,* and *A* are shown below

(5) $$C=\frac{l}{\ln\left(10\right)}\left[\begin{array}{ccccccc} C_{\mathrm{ext}}\left(\lambda_{1},AR_{1},L_{1}\right) & C_{\mathrm{ext}}\left(\lambda_{1},AR_{1},L_{2}\right) & \cdots & C_{\mathrm{ext}}\left(\lambda_{1},AR_{1},L_{I}\right) & C_{\mathrm{ext}}\left(\lambda_{1},AR_{2},L_{1}\right) & \cdots & C_{\mathrm{ext}}\left(\lambda_{1},AR_{J},L_{I}\right)\\ C_{\mathrm{ext}}\left(\lambda_{2},AR_{1},L_{1}\right) & C_{\mathrm{ext}}\left(\lambda_{2},AR_{1},L_{2}\right) & \cdots & C_{\mathrm{ext}}\left(\lambda_{2},AR_{1},L_{I}\right) & C_{\mathrm{ext}}\left(\lambda_{2},AR_{2},L_{1}\right) & \cdots & C_{\mathrm{ext}}\left(\lambda_{2},AR_{J},L_{I}\right)\\ \vdots & \vdots & \vdots & \vdots & \vdots & \ddots & \vdots \\ C_{\mathrm{ext}}\left(\lambda_{K},AR_{1},L_{1}\right) & C_{\mathrm{ext}}\left(\lambda_{K},AR_{1},L_{2}\right) & \cdots & C_{\mathrm{ext}}\left(\lambda_{K},AR_{1},L_{I}\right) & C_{\mathrm{ext}}\left(\lambda_{K},AR_{2},L_{1}\right) & \cdots & C_{\mathrm{ext}}\left(\lambda_{K},AR_{J},L_{I}\right) \end{array}\right],$$

(6) $$P=N{\left[\begin{array}{ccccccc} p\left(AR_{1},L_{1}\right) & p\left(AR_{1},L_{2}\right) & \cdots & p\left(AR_{1},L_{J}\right) & p\left(AR_{2},L_{1}\right) & \cdots & p\left(AR_{j},L_{i}\right)\;\cdots \;p\left(AR_{J},L_{I}\right) \end{array}\right]}^{T},$$

(7) $$A={\left[A\left(\lambda_{1}\right)A\left(\lambda_{2}\right)\cdots A\left(\lambda_{k}\right)\cdots A\left(\lambda_{K}\right)\right]}^{T},$$

here, *i*, *j,* and *k* are integers, and the superscript T denotes the transpose of the vector. *C* is a *K* × (*I* × *J*) matrix consisting of the calculated extinction cross section of Au-Ag alloy nanospheroid; *P* is a (*J* × *I*) × 1 unknown column vector that needs to be solved, and *A* is a *K* × 1 column vector containing the absorbance at different wavelengths. This linear equation can be solved using the non-negative Tikhonov regularization method. Then it can be retrieved to obtain the two-dimensional size distribution and concentration of the particle systems [24,25,26].

## 3. Results and Discussion

**Figure 2:**
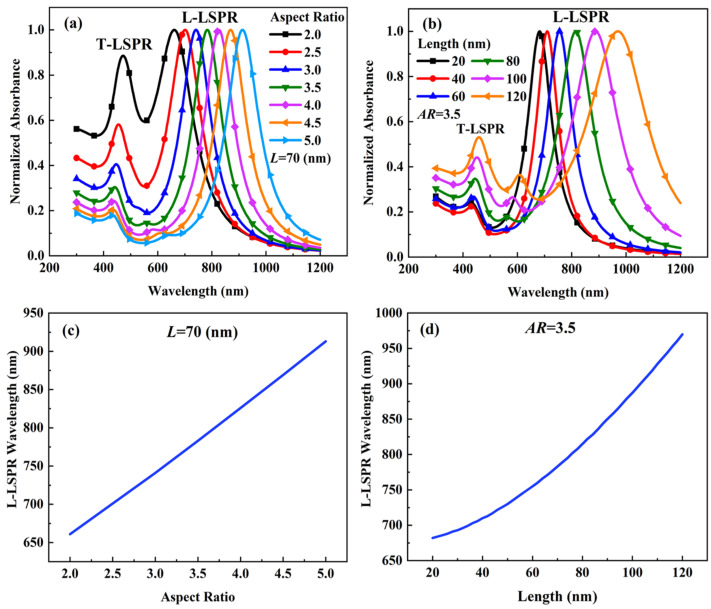
Normalized extinction spectrum (**a**,**b**) and L-LSPR wavelength (**c**,**d**) of monodisperse Au-Ag alloy nanospheroids with different aspect ratios and lengths.

In order to investigate the effects of size on the extinction spectrum of monodisperse Au-Ag alloy nanospheroids, we performed numerical simulations using Equation (1). The results of the simulations are shown in Figure 2. Figure 2a shows the extinction spectrum for Au-Ag alloy nanospheroids for different aspect ratios ranging from 2.0 to 5.0 with an increment step of 0.5. The length *L* is fixed to 70 nm. Figure 2b shows the extinction spectrum for Au-Ag alloy nanospheroids for different lengths L ranging from 20 nm to 120 nm with an increment step of 20 nm. As shown in Figure 2a,b, multiple resonance peaks appear in the extinction spectrum of the Au-Ag alloy nanospheroids when the aspect ratio or the length is increased. The smaller peak is generated by the resonance of free electrons along the transverse direction of the Au-Ag alloy nanospheroids. This peak is called transverse localized surface plasmon resonance (T-LSPR). The major peak is generated by the resonance of free electrons along the longitudinal direction of the Au-Ag alloy nanospheroids. This peak is called longitudinal localized surface plasmon resonance (L-LSPR). The peaks of L-LSPR are used to normalize the extinction spectrum. As shown in Figure 2c,d, the wavelength of L-LSPR increases almost linearly as the aspect ratio increases from 2.0 to 5.0, and exponentially as the length increases from 20 nm to 120 nm. With the increase of aspect ratio or length, a peak redshift phenomenon in the extinction spectrum is observed. This red shift is more obvious in the case of the aspect ratio rather than the length. This makes it possible to retrieve the aspect ratio distribution and length distribution of the polydisperse Au-Ag alloy nanospheroids using the spectral extinction method.

In the case of polydisperse Au-Ag alloy nanospheroids, and due to the large amount of matrix C data required in the retrieval algorithm, it takes a relatively longer time for the T-matrix program to calculate the extinction cross section *C*_ext_. Thus, in order to improve the timeliness of the inversion algorithm program, the extinction cross section *C*_ext_ database of Au-Ag alloy nanospheroids particle systems is established in advance. The related database of extinction cross section of Au-Ag alloy nanospheroids including the wavelength, aspect ratio, and the length is given in Table 1.

Numerical tests are first performed to clarify the accuracy of the retrieval algorithm. We have assumed that the particle size distribution function *p*(*AR_j_*, *L*_i_) satisfies the two-dimensional Gaussian function. The expression of *p*(*AR_j_*, *L*_i_) is:

(8) $$p\left(AR_{j},L_{i}\right)=C\exp\left[-\frac{{\left(AR_{j}-AR_{m}\right)}^{2}}{2\sigma_{\mathrm{AR}}^{2}}-\frac{{\left(L_{i}-L_{m}\right)}^{2}}{2\sigma_{L}^{2}}\right],$$

here, *AR*_m_ is the mean value of the aspect ratio, *σ*_AR_ is the standard deviation of the aspect ratio, *L*_m_ is the mean value of the length of the particle systems, *σ*_L_ is the standard deviation of the length, and *C* is a constant determined by Equation (3). Therefore, the original size parameters of the Au-Ag alloy nanospheroids particle systems need to be set. The wavelength ranges from 400 to 1000 nm, the aspect ratio *AR* ranges from 2.0 to 5.0, the length *L* ranges from 30 to 110 nm, and the particle concentrations *N* is taken as 1 × 10^10^ particles/cm^3^ from the database. The size parameters of the retrieval are set by Equation (8). The mean value of the aspect ratio of the particle systems *AR*_m_ is 3.5. The standard deviation of the aspect ratio $\sigma_{\mathrm{AR}}^{}$ is 0.3, the mean value of the length *L*_m_ of the particle systems is 70 nm and the standard deviation of the length $\sigma_{L}^{}$ is 7 nm. The relative error is used to quantitatively describe the inversion accuracy. It is given by

(9) $$\mathrm{Error}=\left|\frac{\mathrm{Inverted}\;\mathrm{value}\;-\;\mathrm{Original}\;\mathrm{value}}{\mathrm{Original}\;\mathrm{value}}\right|\times 100\%.\;$$

After a large amount of data analysis, it is found that the retrieval relative error is closely related to the step-size of the size distribution. For instance, the aspect ratio *AR* is taken in step-size from 0.1 to 0.5 with an interval of 0.1. The length *L* is taken in step-size of 0.5 to 2.5 nm with an interval of 0.5 nm. So, by varying the aspect ratios and lengths values, the relative error of the retrieval parameters of polydisperse Au-Ag alloy nanospheroids is obtained, as shown in Table 2. Each grid of data in Table 2 represents the mean value of the aspect ratio, the standard deviation of aspect ratio, the mean value of length, the standard deviation of length, and the relative error of inverted concentration from top to bottom. Obviously, when the step-size of the aspect ratio is 0.2 and the step-size of the length is 0.5 nm, the obtained inversion errors are relatively small. It should be noted that this Gaussian distribution is assumed only for numerical tests of the retrieval problem. In fact, it is not necessary to know the size distribution function in advance when using the spectral extinction. Thus, the size distribution of the particles can be represented by any random distribution function.

**Figure 3:**
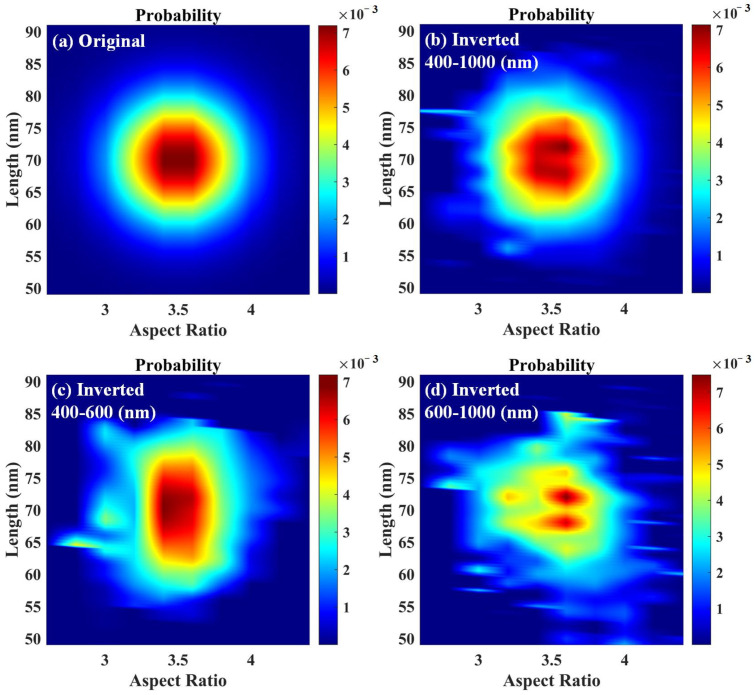
The proportion of the set particle size distribution and the inverted particle size distribution: (**a**) original, (**b**) inverted for a wavelength interval [400–1000 nm], (**c**) inverted for a wavelength interval [400–600 nm], and (**d**) inverted for a wavelength interval [600–1000 nm].

**Figure 4:**
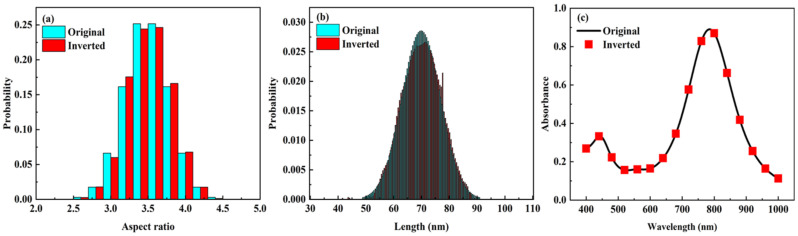
Original and inversion values of (**a**) aspect ratio distribution, (**b**) length distribution, and (**c**) extinction spectra of Au-Ag alloy nanospheroids particles system.

The effect of wavelength range on the retrieval results is further discussed below. A fixed wavelength range was used to find the set of step values with the smallest relative error in the retrieval process. Three wavelength intervals are selected from 400 nm to 1000 nm, i.e., 400–1000 nm, 400–600 nm, and 600–1000 nm. Therefore, the Au-Ag alloy nanospheroids particle systems with two-dimensional Gaussian function of the size distribution are calculated using Equation (8). The original two-dimensional size distribution and the size distribution of the retrieval results with respect to the wavelength intervals are represented in Figure 3. By comparing the original two-dimensional size distribution (Figure 3a) with retrieval results in the wavelength interval from 400 to 1000 nm (Figure 3b), it is found that the aspect ratio of the retrievals is distributed in the range of 3.0 to 4.0, with the highest proportion near the aspect ratio of 3.5. The length of the inversions is distributed in the range of 60 to 80 nm with the highest proportion near the length of 70 nm. The two-dimensional size distribution is similar to the proportion and the retrieval results are good. By comparing the retrieval results in the wavelength interval from 400 to 600 nm (Figure 3c) and in the wavelength interval from 600 to 1000 nm (Figure 3d) with the original two-dimensional size distribution, it is found that the relative error of the retrieval result is relatively large. Therefore, the proportions of the two-dimensional size distributions in Figure 3c,d are further re-stated to obtain the comparison results of the aspect ratio, length, and extinction spectrum, as shown in Figure 4. It can be clearly observed that the distribution percentages of the aspect ratio and the length are very close. Furthermore, the spectrum corresponding to the initially set distributions matches almost exactly with the spectrum reconstructed using the retrieval results, and the relative errors obtained from the retrieval of the aspect ratio, length and extinction spectrum are less than 1%.

However, the measurement errors have an unavoidable influence on the experimental results, so a random noise of 0.1% is added to the extinction spectrum of the particle systems to approximate the experimentally true value. The retrieval results of the initially set two-dimensional size distribution and concentration parameters are obtained with the addition of random noise, as shown in Table 3. The mean value of the aspect ratio, the standard deviation of the aspect ratio, the mean value of the length, the standard deviation of the length and the concentration of the particle systems are 3.52, 0.32, 69.20 nm, 8.10 nm, and 1.0245 × 10^10^ particles/cm^3^ respectively. The corresponding relative errors of the retrieval parameters are 0.57%, 6.67%, 1.14%, 15.71%, and 2.45% respectively. From the retrieval data, it can be seen that the standard deviation of the aspect ratio and the standard deviation of the length obtained with the addition of 0.1% random noise presents a large relative error.

**Figure 5:**
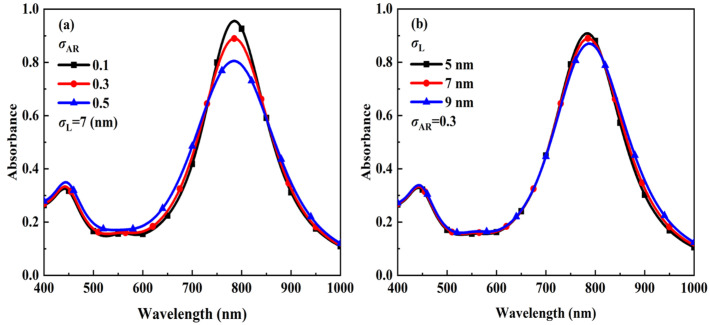
Extinction spectra of polydisperse Au-Ag alloy nanospheroids with different standard deviation of aspect ratio and standard deviation of length. (**a**) Standard deviations of different aspect ratios, (**b**) standard deviations of different lengths.

The reason for the large error may be related to the fact that the extinction spectrum varies less significantly with respect to the aspect ratio standard deviation and to the length standard deviation. Thus, we have analyzed the effect of dimensional standard deviation on the extinction spectrum and the results are shown in Figure 5. For the numerical calculation, the standard deviation of the fixed length is 7 nm and the standard deviations of the aspect ratio are taken as 0.1, 0.3, and 0.5 respectively (Figure 5a). The standard deviation of the fixed aspect ratio is 0.3 and the standard deviations of the length are taken as 5 nm, 7 nm, and 9 nm respectively (Figure 5b). From the results presented in Figure 5, it can be seen that when the standard deviation of the aspect ratio and the standard deviation of the length increase, the change in the extinction spectrum is not obvious leading to an increase in the inversion error. Compared with the standard deviation of the aspect ratio, the standard deviation of the length has less effect on the extinction spectrum and therefore its retrieval error is larger.

## 4. Conclusions

In this paper, using the T-matrix and the non-negative Tikhonov regularization method, a new method based on the extinction spectrum that allows the determination of the two-dimensional size distribution and concentration of the Au-Ag alloy nanospheroids is proposed. The effect of the size-step on the retrieval results is quantitatively analyzed to find the step-size of aspect ratio and length with the smallest retrieval error. Additionally, the suitable wavelength range is selected by performing comparative analysis. Finally, the experimental measurement error of the extinction spectrum is considered. The use of the spectral extinction method offers an attractive alternative to microscopic imaging analysis. While microscopic imaging is extremely valuable for characterization of the shapes made in a synthesis procedure, the spectral extinction method enables a practical method to determine the aspect ratio distribution, length distribution, and number concentration of gold nanorods without microscopic imaging. Thus, this technique presented here allows for easy, straightforward determination of not only the average size but also the size distribution from an extinction spectrum. However, the spectral extinction method also has the limitation that the inversion error will increase when the concentration of the particle system is too high or when there are asymmetric particles in the particle system. Our further study will focus on the application of the spectral extinction method for the characterization of other non-spherical metal nanoparticles such as nanorods, and on the improvement of inverse algorithm to get better inverted results with high accuracy.

## Figures and Tables

**Table 1:** Database range of extinction cross sections of Au-Ag alloy nanospheroids.

Parameters	Ranges	Step-Size
Wavelength (nm)	300–1200	2
Aspect Ratio	1.0–5.0	0.1
Length (nm)	20–120	2

**Table 2:** Retrieval errors corresponding to polydisperse Au-Ag alloy nanospheroids particle systems with different step-size (the mean value of aspect ratio, standard deviation of aspect ratio, mean value of length, standard deviation of length, and concentration).

	Δ*L*	0.5	1.0	1.5	2	2.5
Error (%)						
Δ*AR*						
**0.1**		0.05 2.52 0.10 1.43 0.14	0.04 2.30 0.10 1.59 0.16	0.03 2.50 0.11 1.75 0.17	0.02 1.82 0.05 0.61 0.07	0.03 1.98 0.10 0.57 0.09
**0.2**		0.01 0.85 0.04 0.09 0.07	2.77 27.50 5.52 62.46 12.69	0.02 1.04 0.05 0.32 0.07	0.02 1.18 0.07 0.87 0.10	4.87 52.38 8.80 85.08 21.36
**0.3**		0.01 0.24 0.01 0.92 0.01	0.08 2.58 0.08 1.50 0.08	0.10 2.94 0.09 2.05 0.10	0.09 2.94 0.07 1.79 0.07	0.04 2.13 0.07 0.16 0.06
**0.4**		0.07 1.18 0.13 3.98 0.24	0.07 1.19 0.12 3.64 0.23	0.06 1.28 0.08 2.60 0.14	0.03 1.33 0.01 0.85 0.04	0.02 1.42 0.08 0.60 0.09
**0.5**		0.15 1.90 0.08 1.13 0.24	0.17 2.20 0.04 2.52 0.19	0.17 2.42 0.17 2.89 0.15	0.18 2.24 0.13 0.01 0.34	0.38 10.79 0.07 6.66 0.46

**Table 3:** The results of the inversion are compared with the original results (0.1% random noise).

Parameters	Original	Inverted	Relative Error (%)
*AR*_m_	3.5	3.52	0.57
*σ*_AR_	0.3	0.32	6.67
*L*_m_	70.0 nm	69.20 nm	1.14
*σ*_L_	7.0 nm	8.10 nm	15.71
*N*	1 × 10^10^ particles/cm^3^	1.0245 × 10^10^ particles/cm^3^	2.45
